# A climate- and stage-sensitive stand growth and yield model of natural *Larix gmelinii* forests in northeast China

**DOI:** 10.3389/fpls.2026.1761805

**Published:** 2026-03-04

**Authors:** Lingbo Dong, Xuesong Mei, Zhaogang Liu

**Affiliations:** Key Laboratory of Sustainable Forest Ecosystem Management-Ministry of Education, College of Forestry, Northeast Forestry University, Harbin, China

**Keywords:** aridity index, carbon stock, developmental stage, growth and yield model, natural forest

## Abstract

Understanding the complex interactions between climate change and stand developmental dynamics in forest growth and carbon sequestration is essential for implementing sustainable management under climate change and for supporting China’s dual-carbon goals. Using data from 243 permanent national forest inventory plots (each 0.0667 ha in size), this study developed a climate- and stage-sensitive forest growth and yield model (FGYM) for natural *Larix gmelinii* forests in Northeast China. The model incorporates the De Martonne aridity index (MAI) to represent climatic water availability and the stand developmental stage index, categorized into Stage 1 (early), Stage 2 (middle), and Stage 3 (late), to capture the intrinsic biological progression of forest structure. It simultaneously simulates (i) stand basis structure attributes, (ii) timber yields across different assortments, and (iii) carbon stocks in different tree components and end-use categories. Comparative analyses demonstrated that the stage-sensitive model outperformed the baseline models, revealing pronounced stage- and climate-dependent divergences in stand volume and carbon stock trajectories. For a representative stand [age = 100 years, site class index (SCI) = 16 m], the stage-sensitive model predicted 2.96% higher volume and 3.11% higher carbon stocks at Stage 2, but 15.02% and 15.70% lower values at Stage 3, indicating strong sensitivity to ontogenetic transitions and increasing climatic aridity. Across all combinations of MAI, SCI, and developmental stage, the FGYM consistently captured structural and carbon dynamics that the conventional model did not reproduce. Our findings highlight that integrating both climatic drivers and developmental heterogeneity substantially enhances model accuracy and ecological realism, providing a robust tool for assessing the future productivity and carbon sequestration potential of *L. gmelinii* forests under future climate change scenarios.

## 1 Introduction

Forests are the largest terrestrial carbon reservoirs and play a fundamental role in mitigating global climate change by absorbing atmospheric CO_2_ and stabilizing terrestrial carbon stocks (Harris et al, 2021). According to the Paris Agreement, sustainable forest management and enhanced carbon sequestration in forest ecosystems (Article 5.2) are critical pathways to achieving carbon-neutrality targets. In China, the national “dual carbon” goals, peaking carbon emissions by 2030 and achieving carbon neutrality by 2060, have placed unprecedented emphasis on increasing forest carbon sinks through ecological restoration and precision management. *Larix gmelinii* (Rupr.) is one of the most widespread and ecologically significant species in Northeast China (Dong, 2015; Mamet et al, 2019), forming the dominant forest type in permafrost and cold-temperate regions. These forests not only provide high-quality timber but also represent an essential carbon pool within the Eurasian boreal belt. However, climate change, manifested through increasing temperature, altered precipitation regimes, and more frequent extreme weather events, has profoundly affected the spatial distribution, growth patterns, structural dynamics, and carbon allocation of *L. gmelinii* stands (Carrer and Urbinati, 2006; Zhang et al, 2013; Mamet et al, 2019; Zhang et al, 2022). Because of the long life cycle of trees, developing climate-sensitive models and simulating growth under different climate scenarios are essential for understanding how forest growth and carbon sink processes respond to future climate change and thus for supporting science-based forest management in a changing climate.

Traditional forest growth and yield models have long been used in forest resource assessments and management planning due to their empirical simplicity and robust predictive capabilities (Weiskittel et al., 2009; Lei et al., 2016; Zhang et al., 2022), however, these models typically assume stationary growth–climate relationships and constant site quality, overlooking the nonlinear and stage-dependent nature of forest growth under environmental stress (Zou et al, 2025). Empirical evidence suggests that stand responses to climate variables vary markedly across developmental stages (Boisvenue and Running, 2006; Paquette et al, 2018; Zeller and Pretzsch, 2019): young stands exhibit higher physiological sensitivity to climatic fluctuations, while mature and overmature stands are increasingly constrained by density and structural competition. Thus, ignoring these interactions may lead to biased predictions of stand productivity, biomass accumulation, and carbon sequestration, particularly under future climate scenarios characterized by elevated CO_2_ concentration and warming trends.

Recent advances (e.g., Lei et al., 2016; Kimberley and Watt, 2021; Dong et al., 2025) have sought to integrate climatic variables into forest growth models to enhance their environmental responsiveness. Process-based models (e.g., 3-PG, Biome-BGC) have enhanced our mechanistic understanding of tree growth under climate stressors, but they require extensive physiological and meteorological data (Landsberg et al., 2003; Xie et al., 2017; Yan et al., 2025), which limits their practical application in regional forest management. Alternatively, statistical and hybrid models incorporating site, stand, and climate variables offer a more flexible and data-efficient approach to large-scale growth prediction (West, 2009; Lei et al, 2016; Zou et al, 2025). However, most existing models still treat climate effects as temporally invariant and rarely account for the interactive influence of stand developmental stage and climate variability on forest dynamics. To our best knowledge, Zou et al. (2025) were the first to attempt to incorporate successional stages dummy variables into the volume and carbon storage growth model for subtropical broadleaf secondary forests. They classified the plots into three successional stages using a natural composition index, calculated by multiplying the importance value of each species in the community by its climax-adaptation value. Furthermore, few studies have extended these frameworks to partition total volume and carbon stocks into specific timber assortments and biomass components, which is essential for evaluating the trade-offs between timber production and carbon accumulation under multi-objective management strategies.

To meet the pressing requirements of China’s dual-carbon strategy and to overcome the limitations of existing modeling approaches, this study aims to establish a climate- and stage-sensitive multivariate stand dynamic modeling system for natural *L. gmelinii* forests in Northeast China. To achieve this, we pursued the following specific objectives: (1) to develop a biologically consistent modeling framework by integrating stand structural variables, including stand mean height (TH), quadratic mean diameter (Dg), basal area (BAS), stand density index (SDI), stand volume (VOL), and carbon stock (CAR), with site quality, climatic drivers, and developmental stage effects. (2) to model explicitly the interactive effects between climate and stand development, thereby enabling the simulation of long-term growth trajectories and carbon accumulation. and (3) to couple the system with a provincial standard on the percent of wood assortment (PWA) to allocate total stand volume and carbon stock into different timber assortments (large, medium, small, short, fuelwood, and bark) and tree components (leaf, branch, stem, and root).

## 2 Materials and methods

### 2.1 Study area

**Figure 1:**
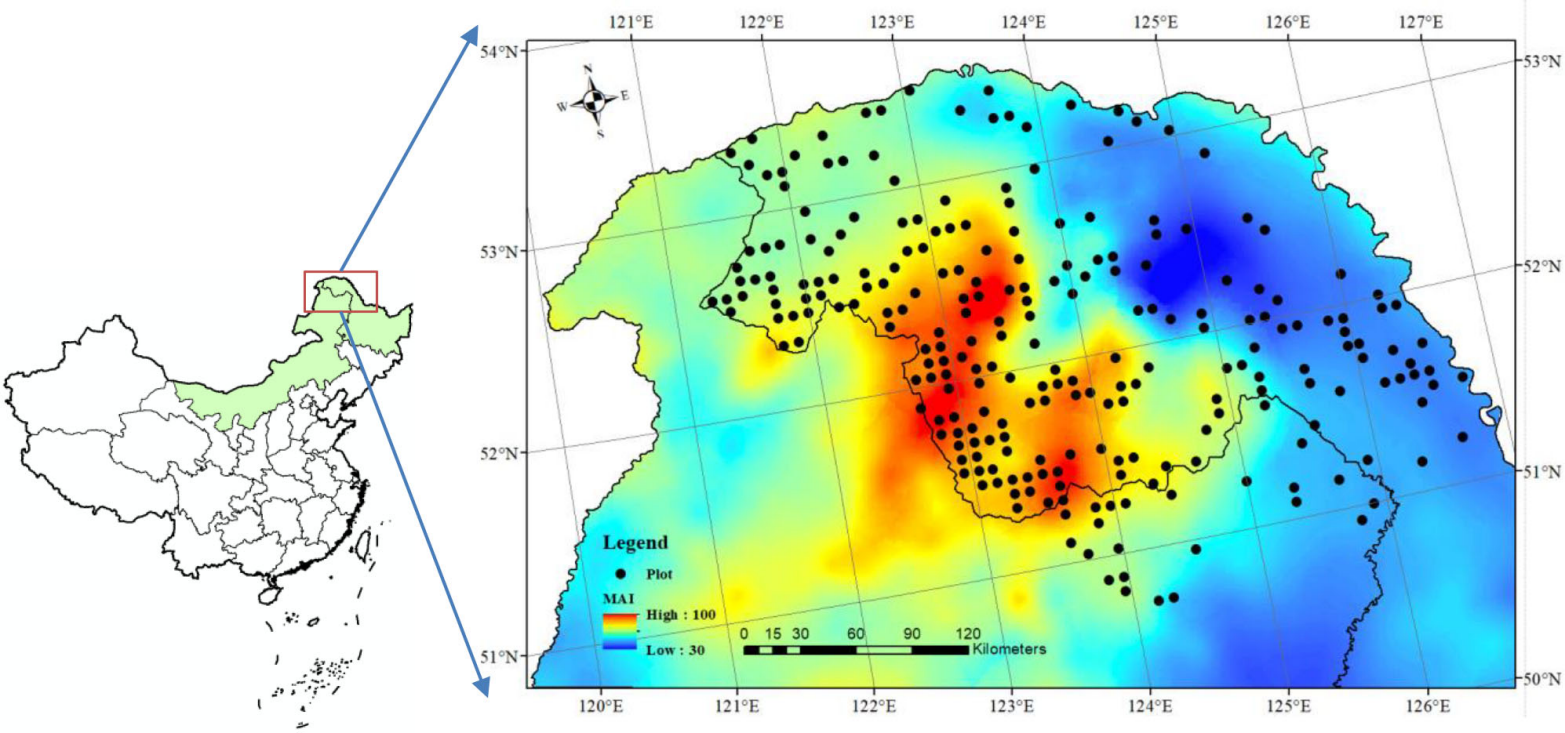
Spatial distribution of sample plots and the De Martonne aridity index.

The study area is located in the northernmost and highest latitude region of China (50°07′02”-53°33′42”N, 121°10′53”-127°01′21”E; **Figure 1**). The terrain is predominantly characterized by hilly and low mountainous landscapes, with an average elevation of approximately 573 m above sea level, rising to a maximum of about 1528m. The region experiences a typical continental monsoon climate, characterized by a mean annual temperature of -2.45°C, an average annual precipitation of approximately 450mm, and a frost-free period lasting 80–100 days. The dominant soil type is dark brown loam, accompanied by smaller proportions of white slurry and meadow soils. The dominant tree species in the region include *Larix gmelinii*, *Pinus sylvestris*, *Betula platyphylla*, and *Populus davidiana*.

### 2.2 Plot data

The data used for this study were obtained from the fixed plots of the 7^th^ National Forest Resource Continuous Inventory (NFI) of China. The plots were established on a systematic 4-km grid, each square in shape and 0.0667 hectares in area. According to the *Technical Provisions for National Forest Resource Continuous Inventory* (State Forestry Administration, 2004), a total of 243 NFI plots were selected from the database, ensuring that *L. gmelinii* accounted for more than 65% of the total stand basal area. For each plot, the recorded variables included geographic coordinates, site factors (e.g., elevation, slope aspect, slope gradient), soil factors (e.g., soil type, soil thickness), stand factors (e.g., stand origin, canopy density), and tree factors (e.g., species, diameter) (**Table 1**). In accordance with the NFI protocol, all trees with a diameter at breast height (DBH) ≥ 5cm were measured and recorded. The measurements included tree species, DBH, vitality status, and relative positions (radius and azimuth).

**Table 1:** Statistics on plots of natural *Larix gmelinii* secondary forests in northeast China.

Variable	Min.	25% quantile	Mean	75% quantile	Max.	Std	CV %
Stand age, T/year	23.0	54.0	87.0	120.0	195.0	40.3	46.4
Quadratic mean diameter, Dg/cm	6.3	10.6	13.1	14.8	23.8	3.4	26.2
Stand mean height, TH/m	4.2	11.5	13.7	16.1	23.0	3.6	26.4
Site class index, SCI/m	6.2	11.6	13.8	16.1	19.7	3.0	21.5
Stand density index, SDI/(trees hm^-2^)	85.3	356.9	585.3	795.8	1190.2	281.2	48.1
Stand volume, SV/(m^3^ hm^-2^)	4.9	48.8	90.6	122.3	265.7	54.0	59.6
Carbon stocks, CS/(ton hm^-2^)	2.0	20.2	38.6	53.2	111.0	23.7	61.4
Mean annual temperature, MAT/°C	-4.9	-3.2	-2.5	-1.7	-0.3	1.0	-39.7
Mean annual precipitation, MAP/mm	331.0	443.3	452.8	470.5	544.2	31.7	7.0
De Martonne aridity index, MAI/(mm/°C)	39.5	52.1	61.7	69.2	95.5	10.6	17.1

The living biomass carbon of each tree, partitioned into root, stem, branch, and leaf components, was estimated using the allometric biomass models developed by Dong (2015) and the carbon concentration coefficients reported by Jia (2014). The adopted carbon fractions were 0.52 for roots, 0.47 for stems, 0.51 for branches, and 0.52 for leaves, respectively. The component-specific carbon stocks were first calculated at the plot level and subsequently converted to a per-hectare level. The corresponding climatic variables for each plot, including mean annual temperature (T) and mean annual precipitation (P), were extracted using the ClimateAP model. In addition, the De Martonne aridity index [MAI = P/(T + 10)] was employed to quantify the site-level aridity condition. Generally, higher MAI values correspond to lower degrees of aridity. Statistics analysis showed that the MAI across the study area ranged from 39.5 to 95.7 mm/°C, with a mean of 61.7 mm/°C and a standard deviation of 10.6 mm/°C (**Table 1**), reflecting a wide range of hydroclimatic conditions.

### 2.3 Forest growth and yield models

#### 2.3.1 Base-FGYM

The base FGYM for natural *L. gmelinii* forests comprises a series of nonlinear equations (Porté and Bartelink, 2002; Weiskittel et al, 2011), namely the site class index model (SCI), stand density index model (SDI), stand basal area model (BAS), stand volume biomass (VOL), stand carbon stock model (CAR), diameter distribution model (DDM) and percent of wood assortment model (PWA). This model system has been widely used for both planted (Porté and Bartelink, 2002; Burkhart, 2008; Lei et al., 2016) and natural forests (Gifford et al., 2023; Zou et al., 2025). As mentioned earlier, the objective of this study is to integrate the dummy variables for the stand development stage and continuous climatic variables into the base FGYM framework.

##### 2.3.1.1 SCI model

To evaluate site conditions, the relationship between stand mean height (TH) and stand age (t) was fitted using a series of commonly used theoretical models (e.g., Mistchlinch, Weibull, Korf, Richard) or empirical models (e.g., Hossfeld; West, 2009). Model performance was assessed based on the adjusted coefficient of determination (adjR^2^), mean absolute error percentage (MAE%), and root mean square error percentage (RMSE%). The results demonstrated that the Richards function significantly outperformed the other models, showing significantly superior goodness of fit.

(1) $$TH=a_{0}{\left[1-\text{exp }\left(-a_{1}t\right)\right]}^{a_{2}}$$

where *TH* is the stand mean height, *t* is the stand age, and $b_{0}$, $b_{1}$ and $b_{2}$ are estimated parameters. Relative to a standard stand, the SCI for a given stand can be expressed as:

(2) $$SCI=TH\frac{{\left[1-\text{exp }(-a_{1}t_{I})\right]}^{a_{2}}}{{\left[1-\text{exp }(-a_{1}t)\right]}^{a_{2}}}$$

where SCI is site class index, $t_{I}$ represents the reference stand age, which was set to 80 years in this study.

##### 2.3.1.2 SDI model

In fully stocked forests, a typical linear relationship between the maximum number of trees per hectare (*N*) and the stand quadratic mean diameter at breast height ( $D_{g}$) can be described on a log-log scale (Equation 3; Reineke, 1933; Kweon and Comeau, 2017). This relationship, known as the maximum stand density line (MSDL) or self-thinning lines in forestry (Reineke, 1933; Comeau et al., 2010; Dong et al., 2025), serves as a benchmark for assessing stand density. Referenced to a standard stand, the SDI for a given stand can be defined as Equation 4. To further characterize the dynamics of SDI, previous studies (Wei, 2003; Chivhenge et al, 2024) reported that SDI generally decreases exponentially with stand age, and this relationship is also significantly influenced by SCI (Equation 5).

(3) $$\ln\left(N\right)=b_{0}+b_{1}\times \ln\left(D_{g}\right)$$

(4) $$SDI=N{\left(D_{0}/D_{g}\right)}^{b_{1}}$$

(5) $$SDI=\left(b_{2}+b_{3}bSCI\right)\left(1-\text{exp}\left(-b_{4}\cdot t\right)\right)$$

where *N* is stand density (trees hm^-2^), *t* is the stand age (years), $D_{g}$ is stand mean DBH (cm), and $D_{0}$ is the mean $D_{g}$ for a standard stand, which was set to 20 cm in this analysis.

##### 2.3.1.3 BAS model

The selection procedure for the base model of stand basal area (BAS) followed a similar approach to that of the SCI model. Based on the evaluation of statistical indicators (adjR2, MAE%, and RMSE%), diagnostic residual plots, and model convergence stability (particularly when considering climate and development stage variables), the Mistchlinch function was ultimately selected to describe the relationship between BAS and stand age (t). Numerous studies (Wei, 2003; Lei et al, 2016) have demonstrated that parameter c is highly sensitive to site conditions, while parameter k is strongly influenced by stand density. Thus, Equation 6 was further extended through a reparameterization process to incorporate the effects of site and density factors (Equation 7).

(6) BAS=c[1−exp (−kt)]

(7) $$BAS=c_{0}SCI^{c_{1}}\left[1-\text{exp }(-k_{0}{\left(SDI/1000\right)}^{k_{1}}t\right]$$

where BAS is the stand basal area (m^2^▪hm^-2^), SCI is the site class index (m), SDI is the stand density index (trees▪hm^-2^), t is the stand age (years), and *c*, *k*, $c_{0}$, $c_{1}$, $k_{0}$ and $k_{1}$ are the estimated parameters.

##### 2.3.1.4 VOL model

Using the estimated BAS and TH, the stand volume per hectare (VOL) was calculated according to Equation 8, which was derived from the concept of artificial form factor (West, 2009; Lei et al, 2016).

(8) $$VOL=d_{0}BAS^{d_{1}}\cdot TH^{d_{2}}$$

where VOL is the stand volume (m^3^▪hm^-2^), BAS is the stand basal area (m^2^▪hm^-2^), and TH is the stand mean height (m), $d_{0}$, $d_{1}$ and $d_{2}$ are the estimated parameters.

##### 2.3.1.5 CAR model

To address the compatibility among different biomass component models, an aggregate additive biomass modeling approach was employed in this analysis. Detailed descriptions about the biomass models are available in Dong et al. (2014). Briefly, the candidate independent variables included commonly used stand attributes, e.g., stand density (N, trees hm^-2^), stand basal area (BAS, m^2^ hm^-2^), stand mean DBH (Dg, cm), stand mean height (TH, m) and stand age (t, year). The dependent variables were the measured living biomass carbon of total ( $W_{t}$), aboveground ( $W_{a}$), belowground ( $W_{b}$), crown ( $W_{c}$), root ( $W_{r}$), stem ( $W_{s}$), branch ( $W_{b}$) and leaf ( $W_{l}$) components, respectively. The general formulations of the aggregative additive biomass models are presented as follows:

(9) $$\begin{array}{r} \text{ln}W_{i}=e_{io}+e_{ij}{\text{lnX}}_{\text{j}}\\ \text{ln}W_{c}=\text{ln}\left(W_{b}+W_{f}\right)\\ \text{ln}W_{a}=\text{ln}\left(W_{s}+W_{b}+W_{f}\right)\\ \text{ln}W_{t}=\text{ln}\left(W_{r}+W_{s}+W_{b}+W_{f}\right) \end{array}$$

where $W_{i}$ represents the living biomass carbon of the *i-*th components, namely total (*t*), aboveground (*a*), root (*b*), stem (*s*), branch (*b*), leaf (*l*) and crown (*c*), respectively.

Correlation analyses indicated that BAS and TH were the two most significant predictors of stand biomass, consistent with the results of the VOL model. Model parameters were estimated using nonlinear seemingly unrelated regression (NSUR), implemented in SAS/ETS, based on 1749 NFI plots. The adjR^2^ for all seven components exceeded 0.90, suggesting high model accuracy and strong predictive performance. Subsequently, the carbon stocks of each component were jointly estimated using the predicted biomass and the measured carbon concentration (Jia, 2014). The estimated parameters of the biomass models from Dong et al. (2014) were illustrated in **Table 2**.

**Table 2:** Parameter estimations and the adjusted R-square (adjR^2^) of aggregative additive biomass models for natural *Larix gmelinii* forests (Dong et al, 2014), where BAS and TH are stand basal area and stand mean height, $e_{i0}$, $e_{i0}$ and $e_{i0}$ are estimated parameters, *ln* is a logarithmic function.

Components	$e_{i0}$	$e_{i1}\left(lnBAS\right)$	$e_{i2}\left(lnTH\right)$	adjR^2^
Root	-1.1579	1.0676	0.5450	0.940
Stem	0.0928	1.0536	0.4248	0.961
Branch	-2.0040	1.0918	0.3687	0.944
Leaf	-1.8690	1.0085	-0.0696	0.978
Crown				0.958
Aboveground				0.963
Total				0.958

##### 2.3.1.6 DDM model

In undisturbed natural forests, a typically negative exponential relationship exists between the number of trees per hectare and the corresponding diameter class (West, 2009):

(10) $$N=f_{0}e^{-f_{1}x}$$

where *N* is the number of trees per hectare in each diameter class (trees▪hm^-2^), x is the midpoint of the diameter class (cm); $f_{0}$ and $f_{1}$ are the estimated parameters. To characterize the variation in tree numbers between consecutive diameter classes, a diameter distribution ratio can be defined as (Equation 11; West, 2009):

(11) $$q=\frac{N_{i-1}}{N_{i}}=\frac{f_{0}e^{-f_{1}x_{i-1}}}{f_{0}e^{-f_{1}x_{i}}}=e^{-f_{1}\left(x_{i-1}-x_{i}\right)}=e^{f_{1}h}$$

where $N_{i}$ is the number of trees per hectare in the *i*-th diameter class, and *h* is the interval width of the diameter class, which was assumed as 2 cm in this analysis.

**Figure 2:**
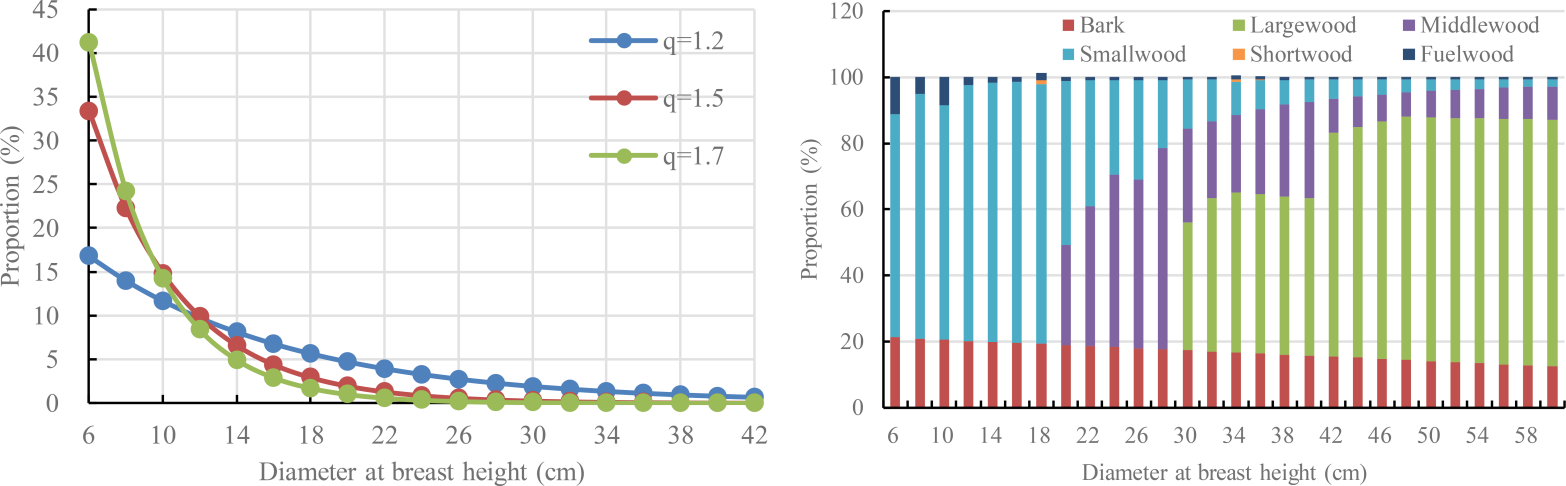
Effects of different *q* values on diameter distribution (left) and variations on the proportion of different wood assortments with varying individual diameter at breast height (right).

The *q*-value is a robust indicator of stand diameter structure; lower q-values (approaching 1.2) reflect flatter diameter distributions with a greater abundance of large-diameter trees. In comparison, higher *q*-values (upper to 1.7) indicate steeper distributions dominated by smaller stems (**Figure 2**). Empirical evidence from undisturbed forests worldwide consistently demonstrates q-values within the range (namely 1.2~1.7; West, 2009), including fir (*Abies nordmanniana*) stands in Turkey (1.34-1.59; Gül et al, 2005), northern hardwood stands in New Hampshire (1.39; Leak, 1996), mixed spruce (*Picea koraiensis*) -fir (*Abies fabri*) forests in northeast China (1.32-1.35; Gong et al, 2009) and mixed broadleaved forests in northeast China (1.23-1.45; Sheng et al, 2024).

With the evolution of forest management philosophies, contemporary management practices increasingly emphasize the cultivation of high-quality and large-diameter timber products. Accordingly, this study hypothesizes that during the natural development of *L. gmelinii* forests, the q-value gradually declines from approximately 1.7 to 1.2, reflecting a progressive increase in the proportion of large-diameter trees within the stand.

##### 2.3.1.7 PWA model

To further quantitatively assess and predict the yield of different timber assortments at various developmental stages of natural *L. gmelinii* forests, this study adopted data from the Heilongjiang Provincial Standard “Yield Rate Table of Main Tree Species for Commercial Forests in Municipal and County Forest Areas (DB23/T870, 2004)”. Specifically, the relationships between timber assortments (e.g., large-diameter timber, medium-diameter timber, small-diameter timber, short-length timber, fuelwood, and bark) and DBH in natural *L. gmelinii* forests are illustrated in **Figure 2**. As shown, large-diameter timber begins to appear at a DBH of approximately 30 cm and increases steadily with further growth. The proportion of medium-diameter timber peaks around 40 cm, whereas that of small-diameter timber reaches its maximum near 18 cm. In contrast, the proportion of bark exhibits a continuous decline as DBH increases.

For analytical convenience, this study categorizes large-, medium-, small- and short-timber as commercial timber, while fuelwood and bark are classified as non-commercial timber. The wood density of all commercial assortments was assumed to be 0.6 t m^-^³, and consistent with that of fuelwood in the non-commercial category (State Forestry and Grassland Administration, 2021). In contrast, the density of bark was assumed to be 0.3 t m^-^³.

#### 2.3.2 Climate-FGYM

The BAS model (Equation 7) not only effectively captures variations in stand density (measured as SDI) and site conditions (measured as SCI), but also serves as an important basis for estimating stand volume (Equation 8). Thus, the BAS model is usually considered as the core component of FGYM (Weiskittel et al, 2011). Numerous studies (Laubhann et al., 2009; Lei et al., 2016; Dong et al., 2025) have consistently demonstrated that climate change has a substantial impact on forest growth processes, particularly in high-latitude regions (e.g., > 50° in this study area). Accordingly, Equation 7 was further reparametrized to develop a climate-sensitive BAS model for natural *L. gmelinii* forests:

(12) $$BAS=c_{0}SCI^{c_{1}}{\left(\frac{1}{MAI}\right)}^{c_{2}}\left[1-\text{exp }(-k_{0}{\left(SDI/1000\right)}^{k_{1}}t\right]$$

where BAS is the stand basal area, SCI is the site class index, SDI is the stand density index, MAI is the De Martonne aridity index, and $c_{0}$, $c_{1}$, $c_{2}$, $k_{0}$ and $k_{1}$ are model parameters to be estimated.

#### 2.3.3 Stage-FGYM

The potential maximums and increment rates of stand volume differ significantly across different developmental stages (Lorimer and Halpin, 2014). Thus, establishing a stage-FGYM not only enhances the simulation accuracy of the overall model but also provides a deeper understanding of the stand growth process. Xu et al. (2024); Wang et al. (2025) systematically compared various methods for classifying developmental stages, including the forestry-based method [e.g., forest factors discrimination (FFD; Lorimer and Halpin, 2014; Xu et al, 2024)], the ecological-based method [e.g., interspecies linkage-optimal segmentation method (ILS; Xu et al, 2024) and TWINSPAN discrimination (TSD; Groenewoud, 1992; Kalantari et al, 2022)], and modern clustering statistics [e.g., agglomerative hierarchical clustering (AHC) and affinity propagation clustering (APC); Wang et al, 2025]. Both studies emphasized that although the classification results obtained using advanced methods (e.g., ecological-based and modern clustering) appeared to be more accurate and reasonable, relatively high anastomosis coefficients (values closer to 1 indicate better agreement) were still observed between these advanced methods and the traditional, simple forestry-based methods. This suggests that simple classification methods, despite their methodological limitations, may still yield comparable practical outcomes in certain contexts.

For natural *L. gmelinii* forests, Wang et al. (2025) divided the entire growth period into three different developmental stages based solely on the Dg discrimination method: Dg<12cm for Stage 1 (79 plots), 12cm≤Dg<18cm for Stage 2 (108 plots), and Dg≥18cm for Stage 3 (56 plots), respectively. To facilitate comparison, we define the condition without distinguishing developmental stages as Stage 0, encompassing all 243 plots. Given stand age remains an essential driver in the FGYM, the corresponding stand age intervals provided for each stage exhibit overlaps (**Supplementary Figure 1**), namely t<100a for Stage 1, 40a≤t<160a for Stage 2, and t≥70a for Stage 3. This is because Dg growth is influenced by multiple factors (e.g., stand density, site factors) beyond age, making such overlap a realistic reflection of stand development dynamics. Nevertheless, t-test results confirmed significant differences in mean stand age among the three stages (*P* < 0.001), with values of 58.52 ± 18.36 years for Stage 1, 83.55 ± 35.94 years for Stage 2, and 131.84 ± 29.30 years for Stage 3, respectively. The anastomosis coefficients between Dg-based FFD and other classification methodologies were evaluated, yielding anastomosis coefficients of 0.52 for TSD, 0.78 for AHC and 0.86 for APC, with higher values indicating greater classification consistency. Given the relatively high consistency, particularly with clustering-based methods, the Dg-based classification scheme was adopted for classifying developmental stages for natural *L. gmelinii* forests in this analysis.

To further quantify the effects of different developmental stages on FGYMs, the dummy-variable technique was employed to construct a stage-sensitive FGYM. Because both the potential maximum value and growth rate of stand volume are sensitive to different developmental stages, the proposed dummy variables were incorporated into both the asymptotic parameter ( $c_{0}$) and the growth rate parameter ( $k_{0}$), respectively.

(13) $$BAS=\left(c_{0}+c_{01}X_{1}+c_{02}X_{2}\right)SCI^{c_{1}}{\left(\frac{1}{MAI}\right)}^{c_{2}}\left[1-\text{exp }(-(k_{0}+k_{01}X_{1}+k_{02}X_{2}){\left(SDI/1000\right)}^{k_{1}}t\right]$$

where BAS is the stand basal area, SCI is the site class index, SDI is the stand density index, MAI is the De Martonne aridity index; *X*_1_ and *X*_2_ are dummy variables for developmental stages, with *X*_1_ = 0 and *X*_2_ = 0 for Stage 1, *X*_1_ = 1 and *X*_2_ = 0 for Stage 2, and *X*_1_ = 1 and *X*_2_ = 1 for Stage 3; $c_{0}$, $c_{01}$, $c_{02}$, $c_{1}$, $c_{2}$, $k_{0}$, $k_{01}$, $k_{02}$ and $k_{1}$ are the estimated parameters.

### 2.4 Evaluation indicators

The parameters for Equation 3 were estimated using linear quantile regression (*rq* function in “quantreg” package) at a 0.99 quantile. In contrast, the parameters for the remaining equations were determined through weighted nonlinear least-squares regression (*nls* function in “stats” package). The weighting functions were defined as the reciprocal form of the corresponding model function (Zeng et al, 2021). Model performances were evaluated using three statistical criteria: adjR^2^ (Equation 14), MAE% (Equation 15), and RMSE% (Equation 16), respectively. A larger $adjR^{2}$ value, together with smaller MAE% and RMSE% values, indicates better model performances.

(14) $$adjR^{2}=1-\frac{\sum_{i=1}^{n}{\left(y_{i}-{\hat{y}}_{i}\right)}^{2}}{\sum_{i=1}^{n}{\left(y_{i}-\bar{y}\right)}^{2}}$$

(15) $$MAE\%=\frac{\sum_{i=1}^{n}\left|\left(y_{i}-{\hat{y}}_{i}\right)\right|}{\sum_{i=1}^{n}\left|y_{i}\right|}\times 100$$

(16) $$RMSE\%=\frac{\sqrt{\frac{\sum_{i=1}^{n}{\left(y_{i}-{\hat{y}}_{i}\right)}^{2}}{n-k-1}}}{\sum_{i=1}^{n}y_{i}/n}\times 100$$

where $y_{i}$ and ${\hat{y}}_{i}$ are the *i*-th observed and estimated values; $\bar{y}$ is the mean value; *n* is the total number of observations; *k* is the number of parameters.

### 2.5 Simulation on the development of stand growth and yield processes

For a specific stand, SCI can be predicted using Equation 2, which was assumed to remain constant throughout the simulation period. The temporal dynamics of key stand variables (e.g., TH, DBH, BAS, NUM, VOL, and CAR) can be predicted following the procedure below.

1. Predict TH at time *t*_2_ using Equation 1:
2. Estimate SDI at time *t*_2_ using Equation 5:
3. Estimate BAS at time *t*_2_ using Equation 7 under the base scenario. When climate effects are considered, Equation 12 should be applied instead; to simultaneously account for developmental stage and climate effects, use Equation 13.
4. Calculate $Dg_{2}$ at time *t*_2_ by combining the definition of BAS ( $BAS_{2}=N_{2}\left(\pi /40000\right)Dg_{2}^{2}$) and the estimated $SDI_{2}$ from step 2, as shown in Equation 4. The resulting formulation for $Dg_{2}$ can be written as (Equation 17):

(17) $$Dg_{2}={\left(\frac{40000D_{0}^{b_{1}}BAS_{2}}{\pi SDI_{2}}\right)}^{\frac{1}{2+b_{1}}}$$

5. Estimate $N_{2}$ (Equation 18) at time *t*_2_ using the definition of BAS ( $BAS_{2}=N_{2}\left(\pi /40000\right)Dg_{2}^{2}$). and the derived $Dg_{2}$ from step 4:

(18) $$N_{2}=\frac{40000BAS_{2}}{\pi Dg_{2}^{2}}$$

6. Predict total VOL (or CAR) using the obtained BAS (Equation 8) and $TH_{2}$ (Equation 9);

7. Estimate carbon stocks for different components (leaf, branch, stem and root) using Equation 9;

8. Allocate the total stand density (N) into different diameter classes ( $N_{i}$) based on Step 5 and Equation 10;

9. Calculate the volume for various assortments (large-, medium-, small-, short-, fuel-wood and bark) and the carbon stocks for different components (leaf, branch, stem and root) using the PWA model, wood density and the estimated $N_{i}$ values from Step 8.

Here, *N*_1_ and *N*_2_ denote the stand densities at time *t*_1_ and *t*_2_, respectively; Dg_2_, BAS_2_, and SDI2 represent the mean DBH, basal area, and stand density index at time *t*_2_.

## 3 Results

### 3.1 Parameter estimations of FGYM models

**Figure 3:**
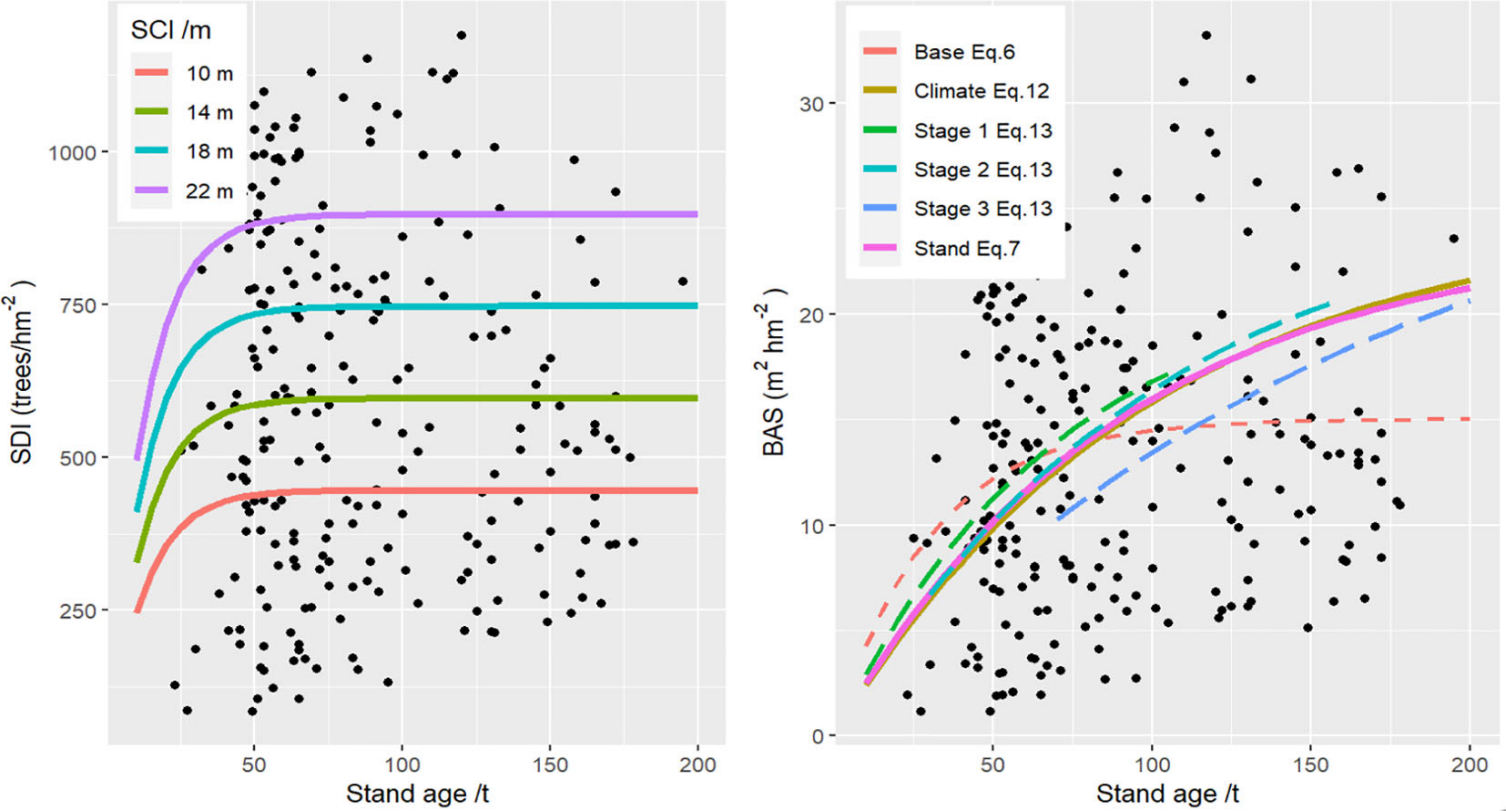
Effects of different site class index (SCI) of the estimated curves between stand age and stand density index (SDI) and the differences on stand basal area (BAS) among different models.

The parameter estimates and goodness-of-fit statistics for the FGYM models were summarized in **Table 3**. For the SCI model, the $adjR^{2}$, MAE%, and RMSE% values were 0.3347, 17.23%, and 21.03%, respectively. Based on Equation 2, the frequency distribution of SCI values at the plot level was illustrated in **Supplementary Figure 2**, showing a mean value of 13.91 m and a standard deviation of 2.93 m. The slope of the SDI model (Equation 3) was significantly shallower (-1.2338) than that of Reineke’s self-thinning law (-1.605; Reineke, 1933). At the plot level, the mean and standard deviation of SDI values were 587.60 ± 282.02 trees hm^-2^ (**Supplementary Figure 3**). Although the SDI dynamic model (Equation 5) exhibited relatively lower accuracy than the other models, with an $adjR^{2}$ of 0.1552, MAE% of 36.53, and RMSE% of 44.28, it still effectively captures the relationship between stand age and SDI, as illustrated in **Figure 3**.

**Table 3:** Parameter estimations and performances of forest growth and yield models for natural *Larix gmelinii* forests, where SCI is site class index, SDI is the stand density index, BAS is the stand basal area, VOL is the stand volume, $adjR^{2}$ is the adjusted R-square, MAE% is the mean percentage of absolute error, and RMSE% is the mean relative root mean square error.

Model	Sub-model	Variable	Estimated value	Std. error	T-value	Pr(>|t|)	$adjR^{2}$	MAE%	RMSE%
SCI	Equation 1	$a_{0}$	22.1148	9.0345	2.448	0.0151	0.3347	17.23	21.03
		$a_{1}$	0.0046	0.0843	0.552	0.0582			
		$a_{2}$	0.4059	0.1648	2.462	0.0145			
SDI	Equation 3	$b_{0}$	10.7253	0.7541		<0.0001	–	–	–
		$b_{1}$	-1.2338	0.1886		<0.0001			
	Equation 5	$b_{2}$	77.7472	8.0134	9.700	0.0333	0.1552	36.53	44.28
		$b_{3}$	37.3025	5.7179	6.524	<0.0001			
		$b_{4}$	0.0834	0.0486	1.715	0.0877			
BAS	Equation 6	*C*	14.7891	0.8108	18.239	<0.0001	0.0407	42.68	51.27
		*K*	0.0350	0.0082	4.271	<0.0001			
	Equation 7	$c_{0}$	6.0329	1.0107	5.969	<0.0001	0.8815	14.45	18.95
		$c_{1}$	0.5166	0.0618	8.358	<0.0001			
		$k_{0}$	0.0317	0.0027	11.606	<0.0001			
		$k_{1}$	1.9227	0.0810	23.738	<0.0001			
	Equation 12	$c_{0}$	22.8911	7.5749	3.022	0.0028	0.8906	13.94	18.09
		$c_{1}$	0.3015	0.0667	4.521	<0.0001			
		$c_{2}$	0.4972	0.0593	8.391	<0.0001			
		$k_{0}$	0.0276	0.0024	11.524	<0.0001			
		$k_{1}$	1.8271	0.0760	24.049	<0.0001			
	Equation 13	$c_{0}$	12.5211	4.0114	3.121	0.0020	0.9039	12.78	16.57
		$c_{01}$	2.2253	1.2178	1.827	0.0689			
		$c_{02}$	3.9381	1.7899	2.200	0.0288			
		$c_{1}$	0.5966	0.0679	8.782	<0.0001			
		$c_{2}$	0.2476	0.0636	3.891	0.0001			
		$k_{0}$	0.0362	0.0050	7.195	<0.0001			
		$k_{01}$	-0.0109	0.0046	-2.387	0.0178			
		$k_{02}$	-0.0207	0.0046	-4.457	<0.0001			
		$k_{1}$	1.6959	0.0803	21.126	<0.0001			
VOL Equation8	Equation 8	$d_{0}$	2.9095	0.1771	16.43	<0.0001	0.9806	6.00	7.60
		$d_{1}$	1.0096	0.0140	71.97	<0.0001			
		$d_{2}$	0.3066	0.0237	12.92	<0.0001			

The accuracy of the base BAS model (Equation 6) was relatively low, with an $adjR^{2}\;\text{of}\;0.0407$. However, a significant improvement in $adjR^{2}$ (0.8815) could be observed when both SCI and SDI were incorporated into the model (Equation 7). Further inclusion of climate-related variable (Equation 12) and stage-related variable (Equation 13) enhanced the model accuracy by approximately 1.03% and 2.54% in $adjR^{2}$, respectively, when compared to the stand-related BAS model (Equation 7). Correspondingly, notable reductions in MAE% and RMSE% were also achieved for Equation 12, 13, showing decreases of 3.53% and 11.57% in MAE%, and 4.54% and 12.56% in RMSE%, respectively. As illustrated in **Figure 3**, substantial differences in the estimated BAS were evident among the four BAS models.

**Figure 4:**
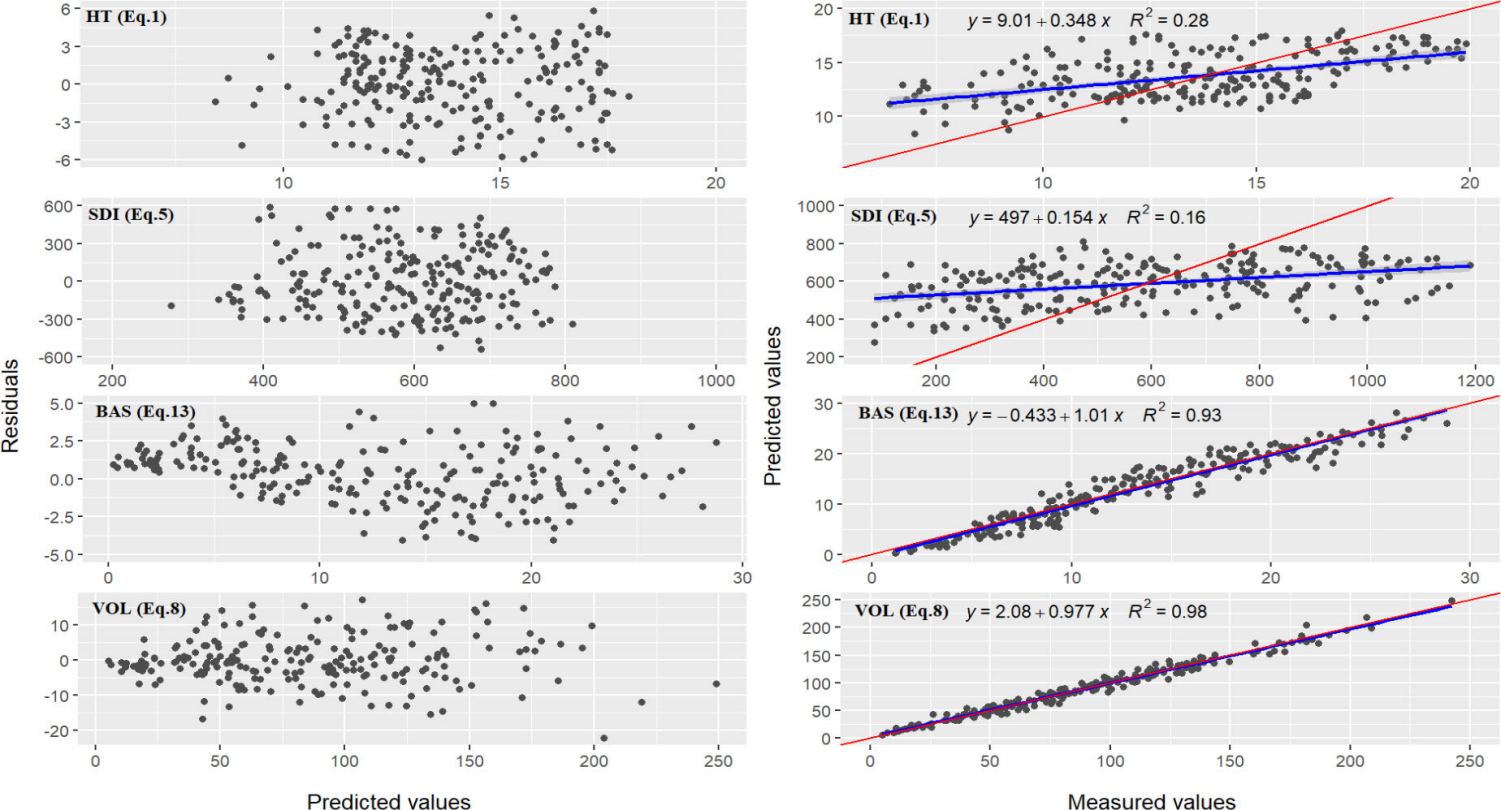
The residuals distributions (left) and the relationships (right) between measured- and predicted-values for stand mean height (TH), stand density index (SDI), stand basal area (BAS), and stand volume (VOL) for natural *Larix gmelinii* forests.

The VOL model exhibited robust statistical performance, with an $adjR^{2}$ of 0.9806, MAE% of 6.00 and RMSE% of 7.60, collectively indicating a high level of model accuracy. **Figure 4** presented the residual distributions and the correlations between the measured and predicted values for the TH, SDI, BAS, and VOL models. These graphical assessments confirmed that all models satisfied the fundamental prerequisites for biometric modeling, e.g., the assumption of homoscedasticity.

### 3.2 Simulations with the developed FGYM models

**Figure 5:**
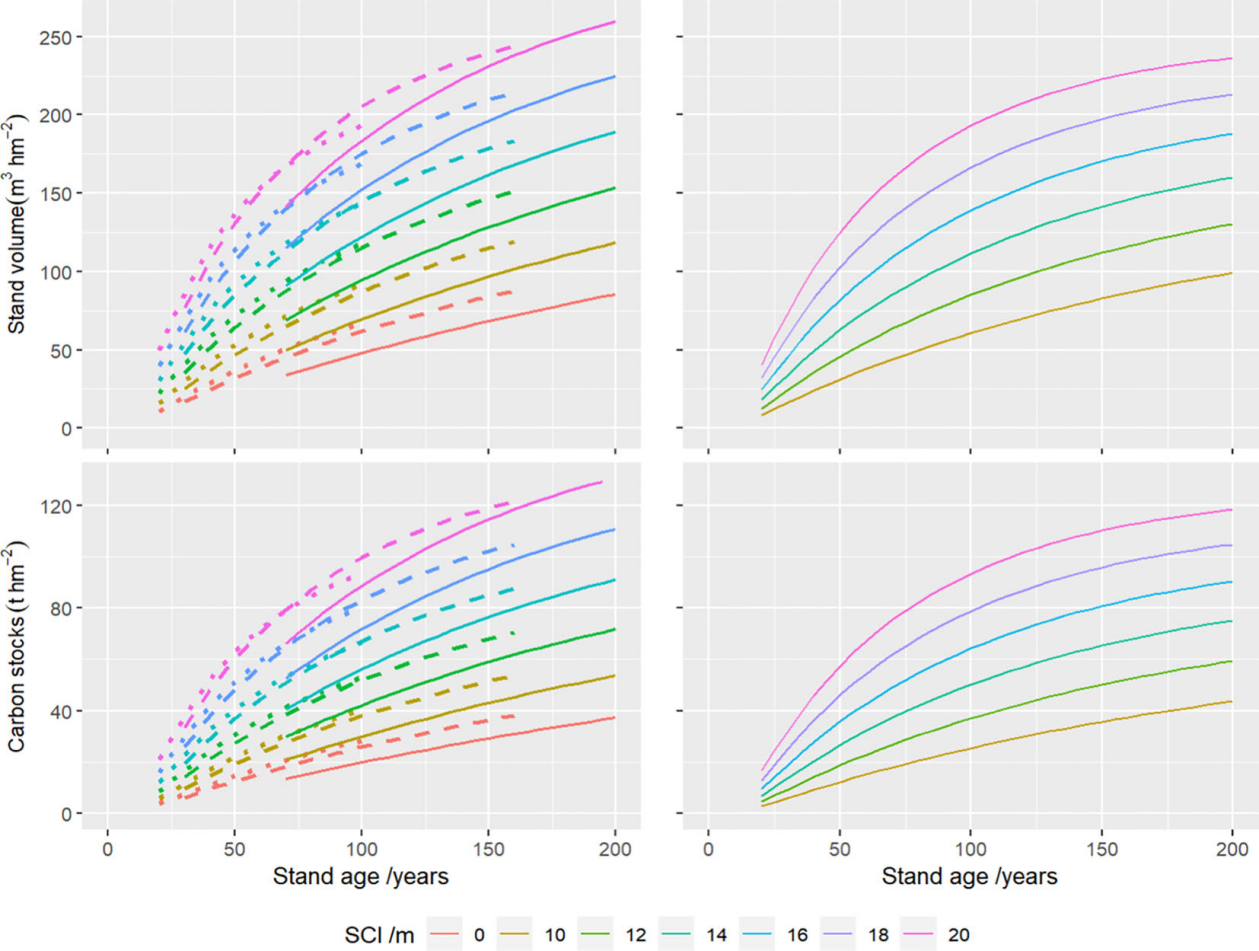
Growth processes of stand volume and carbon stocks using the developed forest growth and yield models with (left) and without (right) developmental stages, where SCI is the stand site class index, the dotted, dashed, and solid lines represent the stage 1, stage 2 and stage 3 of natural *Larix gmelinii* forests in northeast China.

The developed FGYM enables the simulation of (i) stand structure attributes, including Dg, TH, BAS, NUM and SDI); (ii) volume yields across different timber assortments (large-, medium-, small-, short-, fuel-wood and bark); and (iii) carbon stocks in various tree components (leaf, branch, stem and root) and end-use categories (commercial- and noncommercial- wood) under different combinations among MAI, SCI and stand age (see details from Appendix). Comparative analyses between the Stage-FGYM and the Base-FGYM revealed significant divergences in both stand volume and carbon stock trajectories (**Figure 5**). For a representative stand (age=100 years, SCI = 16m), the Base-FGYM predicted baseline estimates of 126.42 m^3^ hm^-2^ (volume) and 57.80 t hm^-2^ (carbon), while the Stage-FGYM simulations indicated clearly stage-dependent variations: at Stage 2 (Dg=16 cm), volume and carbon stocks increased by approximately 2.96% and 3.11% respectively, whereas they decreased by 15.02% and 15.70% at Stage 3 (Dg=20 cm), demonstrating the model’s sensitivity to ontogenetic transitions and developmental phase shifts.

**Table 4** provides an example illustrating the potential effects of different MAIs (50, 60, and 70), SCIs, and development stages on stand characteristics, volume, and carbon stock when stand age is fixed at 80 years. For a specific MAI, Dg, BAS, VOL, and CAR all exhibited significant increases with rising SCI, irrespective of development stages, whereas NUM was strongly correlated with the developmental stage. In contrast, for any specific SCI, Dg, BAS, VOL, and CAR showed marked declines as MAI increased. Across all combinations between MAI and SCI, predicted values of Dg, BAS, VOL, and CAR at Stage 1 were notably larger than those obtained at Stage 0 (baseline, without development stage information), while results of Stage 2 and Stage 3 were generally comparable. However, estimates at Stage 3 were consistently and significantly lower than those of Stage 0, indicating the influence of ontogenetic adjustment. Interestingly, variations of NUM across different development stages exhibited an entirely opposite trend compared with the other variables.

**Table 4:** Simulations on stand characteristics, stand volume and stand carbon at 80 years for different combinations among climate scenarios, development stages and site class index (SCI) of natural *Larix gmelinii* forests in northeast China, where Base is the base scenario, MAI is the De Martonne aridity index, Dg is quadratic mean diameter at breast height, BAS is stand basal area, NUM is stand density, C_Comm is commercial wood, C_Non is non-commercial wood; Stage 0 denotes stage-independent simulations, and Stage 1–3 denote stage-dependent simulations that were classified based on Dg discrimination method (Wang et al., 2025), namely Dg<12cm for Stage 1, 12cm≤Dg<18cm for Stage 2, and Dg≥18cm for Stage 3.

Scenario	Stage	SCI /m	Stand status			Stand volume (m^3^ hm^-2^)							Stand carbon (t hm^-2^)						
			Dg /cm	BAS /(m^2^ hm^-2^)	NUM /(trees hm^-2^)	Total	Bark	Large	Middle	Small	Short	Fuel	Total	Root	Stem	Branch	Leaf	C_Non	C_Comm
Base	0	12	12.1	11.2	964	71.2	14.6	0.2	1.6	50.0	0.0	4.8	30.5	8.3	19.0	2.4	0.8	3.6	15.4
	0	16	15.5	17.4	916	121.6	25.0	0.4	2.7	85.3	0.0	8.1	55.3	15.6	34.2	4.3	1.2	6.5	27.8
	0	20	17.4	23.2	973	174.5	35.9	0.6	3.9	122.4	0.1	11.7	83.1	24.0	51.1	6.4	1.6	9.7	41.5
MAI50	0	12	13.1	11.8	877	75.6	15.5	0.3	1.7	53.0	0.0	5.1	32.4	8.9	20.2	2.6	0.8	3.8	16.4
	0	16	16.5	18.2	848	127.6	26.2	0.4	2.9	89.6	0.0	8.5	58.2	16.4	36.0	4.5	1.2	6.8	29.2
	0	20	18.6	24.4	900	183.2	37.7	0.6	4.1	128.6	0.1	12.3	87.5	25.3	53.8	6.8	1.6	10.2	43.6
	1	12	14.7	12.9	759	82.7	17.0	0.3	1.8	58.1	0.0	5.5	35.7	9.7	22.2	2.8	0.9	4.2	18.0
	1	16	17.7	19.2	782	134.2	27.6	0.5	3.0	94.2	0.0	9.0	61.4	17.3	38.0	4.8	1.3	7.2	30.8
	1	20	19.0	24.8	874	186.6	38.4	0.6	4.2	131.0	0.1	12.5	89.2	25.8	54.9	6.9	1.7	10.4	44.5
	2	12	13.3	12.0	860	76.5	15.7	0.3	1.7	53.7	0.0	5.1	32.9	9.0	20.5	2.6	0.8	3.9	16.6
	2	16	17.1	18.7	814	131.0	26.9	0.4	2.9	91.9	0.0	8.8	59.8	16.9	37.0	4.7	1.3	7.0	30.0
	2	20	19.6	25.5	839	191.4	39.3	0.6	4.3	134.3	0.1	12.8	91.6	26.5	56.3	7.1	1.7	10.7	45.7
	3	12	9.7	9.4	1280	59.6	12.3	0.2	1.3	41.9	0.0	4.0	25.3	6.9	15.8	2.0	0.6	3.0	12.8
	3	16	13.2	15.3	1120	107.2	22.0	0.4	2.4	75.2	0.0	7.2	48.5	13.7	30.0	3.8	1.0	5.7	24.3
	3	20	16.2	21.9	1066	164.7	33.9	0.6	3.7	115.6	0.1	11.0	78.3	22.6	48.2	6.0	1.5	9.1	39.1
MAI60	0	12	12.1	11.2	964	71.2	14.6	0.2	1.6	50.0	0.0	4.8	30.5	8.3	19.0	2.4	0.8	3.6	15.4
	0	16	15.3	17.2	932	120.3	24.7	0.4	2.7	84.4	0.0	8.0	54.7	15.4	33.9	4.3	1.2	6.4	27.5
	0	20	17.2	23.0	989	172.7	35.5	0.6	3.9	121.2	0.1	11.6	82.3	23.8	50.6	6.4	1.5	9.6	41.0
	1	12	13.8	12.3	820	78.8	16.2	0.3	1.8	55.3	0.0	5.3	33.9	9.3	21.1	2.7	0.9	4.0	17.1
	1	16	16.6	18.3	845	127.9	26.3	0.4	2.9	89.8	0.0	8.6	58.4	16.5	36.1	4.6	1.2	6.8	29.3
	1	20	17.9	23.7	944	177.8	36.6	0.6	4.0	124.8	0.1	11.9	84.8	24.5	52.2	6.6	1.6	9.9	42.3
	2	12	12.5	11.4	928	72.9	15.0	0.2	1.6	51.2	0.0	4.9	31.2	8.5	19.5	2.5	0.8	3.7	15.8
	2	16	16.1	17.8	879	124.8	25.7	0.4	2.8	87.6	0.0	8.3	56.9	16.0	35.2	4.4	1.2	6.7	28.5
	2	20	18.5	24.3	907	182.4	37.5	0.6	4.1	128.0	0.1	12.2	87.1	25.2	53.6	6.7	1.6	10.1	43.4
	3	12	9.1	8.9	1382	56.8	11.7	0.2	1.3	39.9	0.0	3.8	24.0	6.5	15.0	1.9	0.6	2.8	12.2
	3	16	12.4	14.6	1210	102.1	21.0	0.3	2.3	71.7	0.0	6.8	46.1	13.0	28.5	3.6	1.0	5.4	23.2
	3	20	15.2	20.9	1151	157.0	32.3	0.5	3.5	110.2	0.1	10.5	74.4	21.5	45.8	5.7	1.4	8.7	37.1
MAI70	0	12	11.4	10.6	1044	67.8	13.9	0.2	1.5	47.6	0.0	4.5	28.9	7.9	18.0	2.3	0.7	3.4	14.6
	0	16	14.4	16.4	1009	114.5	23.5	0.4	2.6	80.3	0.0	7.7	51.9	14.6	32.2	4.0	1.1	6.1	26.1
	0	20	16.1	21.9	1071	164.3	33.8	0.6	3.7	115.3	0.1	11.0	78.1	22.5	48.0	6.0	1.5	9.1	39.0
	1	12	13.1	11.9	875	75.7	15.6	0.3	1.7	53.1	0.0	5.1	32.5	8.9	20.2	2.6	0.8	3.8	16.4
	1	16	15.7	17.5	902	122.8	25.2	0.4	2.7	86.2	0.0	8.2	55.9	15.8	34.6	4.4	1.2	6.5	28.1
	1	20	16.9	22.7	1008	170.7	35.1	0.6	3.8	119.8	0.1	11.4	81.2	23.5	50.0	6.3	1.5	9.5	40.5
	2	12	11.9	11.0	991	70.0	14.4	0.2	1.6	49.1	0.0	4.7	29.9	8.2	18.7	2.3	0.8	3.5	15.1
	2	16	15.2	17.1	938	119.8	24.6	0.4	2.7	84.1	0.0	8.0	54.5	15.4	33.7	4.2	1.2	6.4	27.3
	2	20	17.5	23.3	968	175.1	36.0	0.6	3.9	122.9	0.1	11.7	83.4	24.1	51.3	6.5	1.6	9.7	41.6
	3	12	8.6	8.6	1475	54.5	11.2	0.2	1.2	38.3	0.0	3.6	23.0	6.3	14.4	1.8	0.6	2.7	11.7
	3	16	11.8	14.0	1291	98.0	20.2	0.3	2.2	68.8	0.0	6.6	44.1	12.4	27.4	3.4	0.9	5.2	22.2
	3	20	14.4	20.1	1229	150.7	31.0	0.5	3.4	105.8	0.1	10.1	71.3	20.6	43.9	5.5	1.3	8.3	35.6

## 4 Discussions

### 4.1 Climate influences on stand growth and yield

Our modeling results clearly demonstrate that climatic variables are the primary drivers of stand structural dynamics, volume yield, and carbon accumulation in natural *Larix gmelinii* forests. As expected, for a given MAI, increasing SCI significantly enhanced Dg, BAS, VOL, and CAR across all developmental stages. This finding aligns with previous studies, which have shown that favorable climatic and edaphic conditions promote higher forest productivity and biomass accumulation in boreal ecosystems (Wang et al., 2011; Dong et al., 2024). Furthermore, the necessity of integrating climatic factors into empirical growth and yield models has been repeatedly emphasized (Lei et al., 2016; Trasobares et al., 2016; Zou et al., 2025), given that temperature, precipitation, and the corresponding drought indices strongly influence productivity trends and their spatiotemporal variability.

Interestingly, our simulations revealed that, for a fixed SCI, higher assumed MAI values (representing more humid conditions) were associated with lower Dg, BAS, VOL, and CAR. This counterintuitive result suggests that under stable stand structural conditions, a more humid climate (i.e., higher MAI) does not necessarily lead to greater biomass or carbon storage. Possible explanations include reduced solar radiation, lower temperature-driven growth rates and nutrient leaching from soils under excessively moist conditions (Mirabel et al, 2023), which collectively limit forest productivity. It reinforces that management goals focused solely on increasing increment rates without considering climatic or structural constraints may compromise long-term sequestration efficiency.

### 4.2 Developmental stage modulates climate–growth responses

A notable strength of this study is its explicit accounting for the interaction between developmental stage and climate. The results reveal stage-dependent divergences in the trajectories of stand growth and carbon accumulation. Specifically, the predicted Dg, BAS, VOL, and CAR at Stage 1 were significantly higher than those of the stage-independent baseline (Stage 0), whereas Stage 3 exhibited a pronounced decline. These patterns reflect ontogenetic shifts in resource allocation, physiological efficiency, and self-thinning intensity throughout stand development (McMillan et al, 2008). Early-stage stands typically display higher responsiveness to favorable climatic conditions, while mature stands tend to experience physiological saturation and competitive constraints (Del Río et al, 2014).

The observed interactive effects suggest that the developmental stage acts as a critical mediator of climatic sensitivity. Similar findings have been reported in boreal and temperate forests, where stand age and structure strongly modify climate–growth relationships (McMillan et al, 2008; Wang et al, 2011). For example, Zhu et al. (2019) found that the climate sensitivity of net primary productivity (NPP) was greatest during dense stem phases following regeneration, illustrating that structural dynamics influence climate responsiveness. Our results thus confirm that growth and carbon sequestration in natural *L. gmelinii* are not static processes but evolve dynamically through developmental transitions.

### 4.3 Implications for carbon sequestration and yield under the dual-carbon framework

The climate-stage interaction captured in our model offers valuable insights for optimizing forest management in line with China’s dual-carbon goals. The model indicates that younger and mid-developmental stages (Stages 1–2) exhibit higher growth and carbon accumulation potential compared with older stages (Stage 3), suggesting that stage-sensitive management interventions (e.g., density regulation) could maximize both timber yield and carbon sequestration. Conversely, older stands (Stage 3) demonstrated reduced growth and storage potential, implying the necessity of timely regeneration or structural rejuvenation to maintain sink strength. These findings align with evidence that forest carbon sequestration efficiency declines with stand age and that active management can extend the duration of high sequestration phases (Keenan, 2015; Li et al, 2025). In addition, partitioning total stand volume into assortments (large, medium, small, short, fuelwood, bark) and biomass carbon into different components (leaf, branch, stem and root) further supports rational planning of subsequent utilization and potential economic returns (Pukkala, 2018; Lin et al, 2024). This framework provides a foundation for adaptive, stage-sensitive decision-making that balances ecological and economic objectives in the context of climate change.

### 4.4 Limitations and future research

Despite the progress made, several limitations warrant attention. First, the model currently uses prescribed MAI values, which may not fully capture the effects of spatial and temporal variability in moisture conditions on stand growth and carbon accumulation. Second, disturbance factors (e.g., pests, fires) remain implicit, although such processes are expected to intensify under future climate change (Hu et al., 2012; Seidl et al., 2017). Third, while the framework is calibrated for *L. gmelinii* in Northeast China, recalibration for species- and region-specific applications is essential before its broad application. Future work should focus on integrating process-based submodules to capture physiological mechanisms and disturbance regimes, as well as employing remote-sensing products for large-scale validation. The development of coupled empirical–mechanistic frameworks will improve predictive accuracy and support more robust scenario analyses. Additionally, incorporating adaptive silvicultural options, such as variable retention thinning and assisted migration, could further enhance the model’s applicability and management decision-making.

## 5 Conclusion

Our climate- and developmental stage-sensitive modeling framework for natural *L. gmelinii* forests demonstrates that a higher site condition index (SCI) consistently enhances stand volume and carbon stocks, with VOL reaching 126 m³ ha^-^¹ and CAR reaching 58 t ha^-^¹ at age 100 years under typical conditions. In contrast, excessively humid climates (higher MAI) reduced VOL and CAR by up to 15% at Stage 3, highlighting the nonlinear effects of moisture on forest productivity. Stage-dependent dynamics further revealed that early- and mid-developmental stages (Stages 1–2) exhibited the highest potential for volume accumulation and carbon sequestration, whereas mature stands (Stage 3) were more constrained by structural limitations. By integrating timber assortment allocation and carbon partitioning among different components, the model provides a practical tool for optimizing forest yield and carbon storage. Overall, these results highlight the importance of considering both climatic variability and ontogenetic stage in predictive models, providing a scientific basis for stage-sensitive, climate-adaptive management strategies that align with China’s dual-carbon objectives.

## Data Availability

The original contributions presented in the study are included in the article/**Supplementary Material**. Further inquiries can be directed to the corresponding author.
