# Supplementary material for: A climate- and stage-sensitive stand growth and yield model of natural Larix gmelinii forests in northeast China
Source: Front Plant Sci. 2026 Mar 4;17:1761805. doi: 10.3389/fpls.2026.1761805 (PMC12996187; doi:10.3389/fpls.2026.1761805)
Supplement: Supplementary file 2 [file DataSheet1.docx]

**A climate- and stage-sensitive stand growth and yield model of natural *Larix gmelinii* forests in northeast China**

Lingbo Dong^a^, Fengri Li^a^, Zhaogang Liu^a^

^a^ Key Laboratory of Sustainable Forest Ecosystem Management-Ministry of Education, College of Forestry, Northeast Forestry University, Harbin 150040, China; [farrell0503@126.com](mailto:farrell0503@126.com);

^b^ College of Forestry, Oregon State University, Corvallis 97331, OR, USA; [woodam.chung@oregonstate.edu](file:///E:\国外工作\0-Error%20structure\woodam.chung@oregonstate.edu);

* Corresponding author: [lzg19700602@163.com](mailto:lzg19700602@163.com) (Z. Liu)


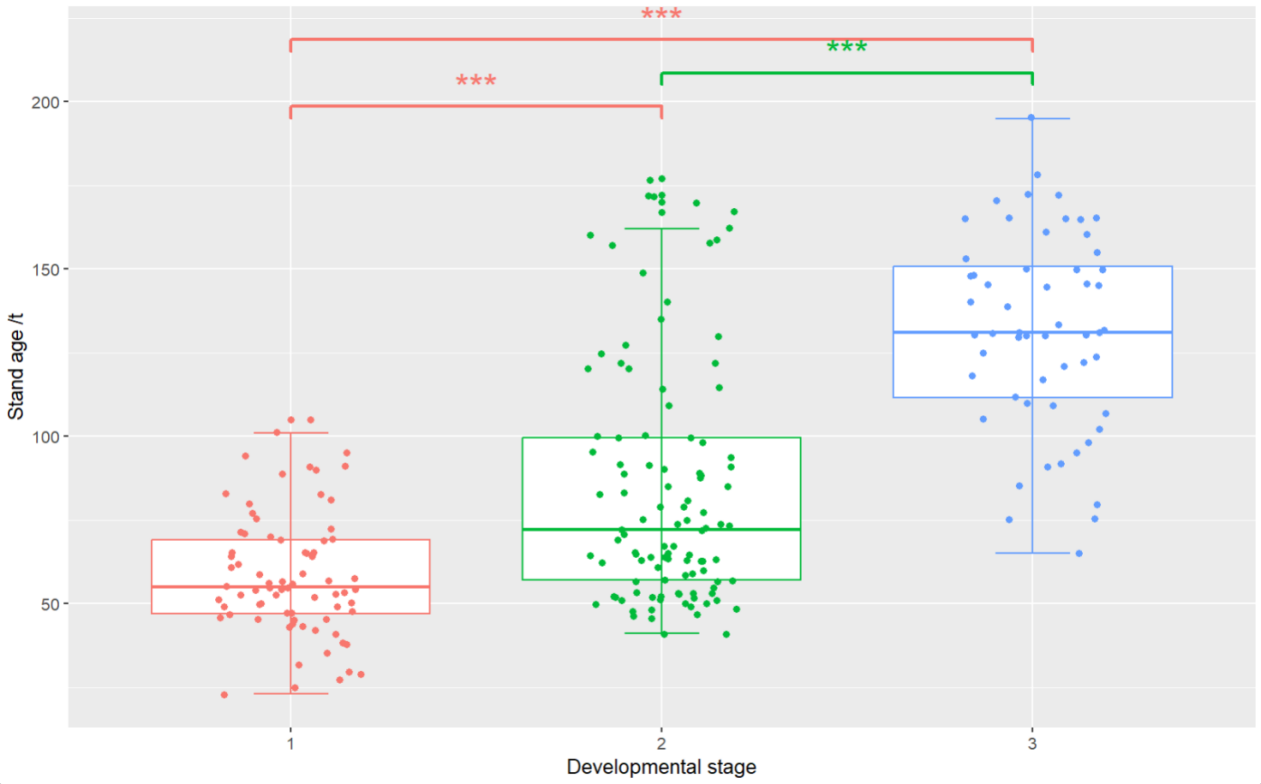


**Fig.S1** Differences on stand ages among different developmental stages of natural *Larix gmelinii* forests, where *** indicated a 0.001 significance level.


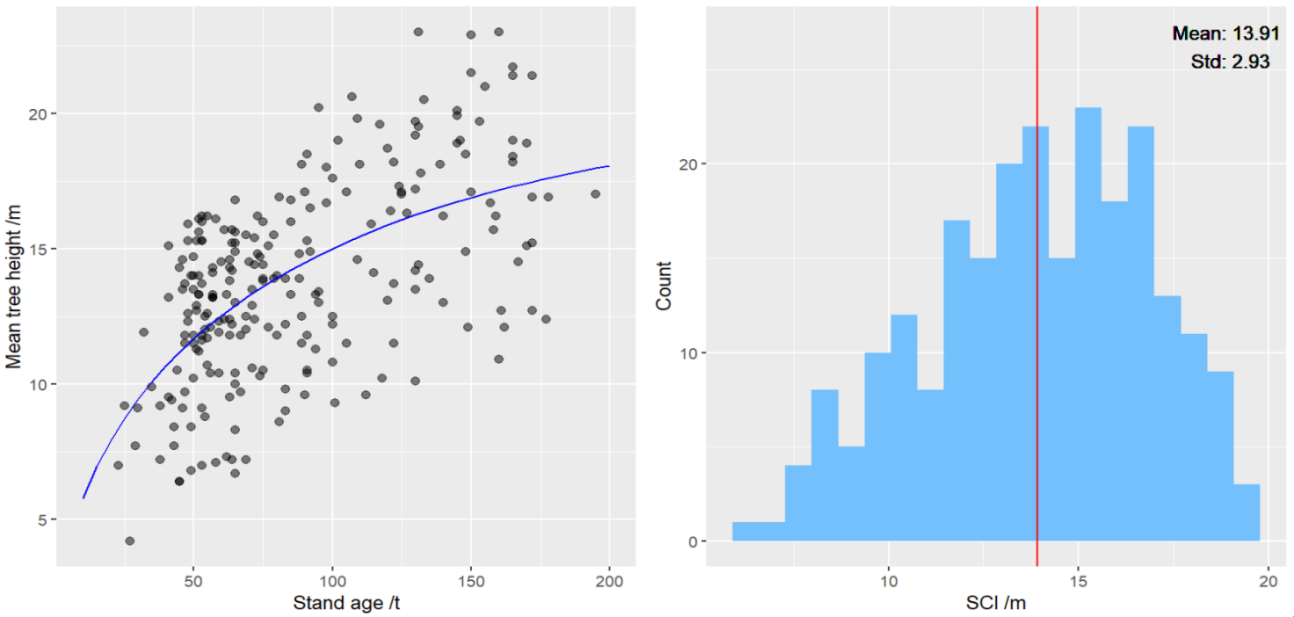


**Fig. S2** The fitted mean tree height *vs* stand ages curve (blue; left) and the corresponding frequency distribution of stie class index (SCI; right) for natural Larix *gmelinii* forests, where the red line is the mean value of SCI.


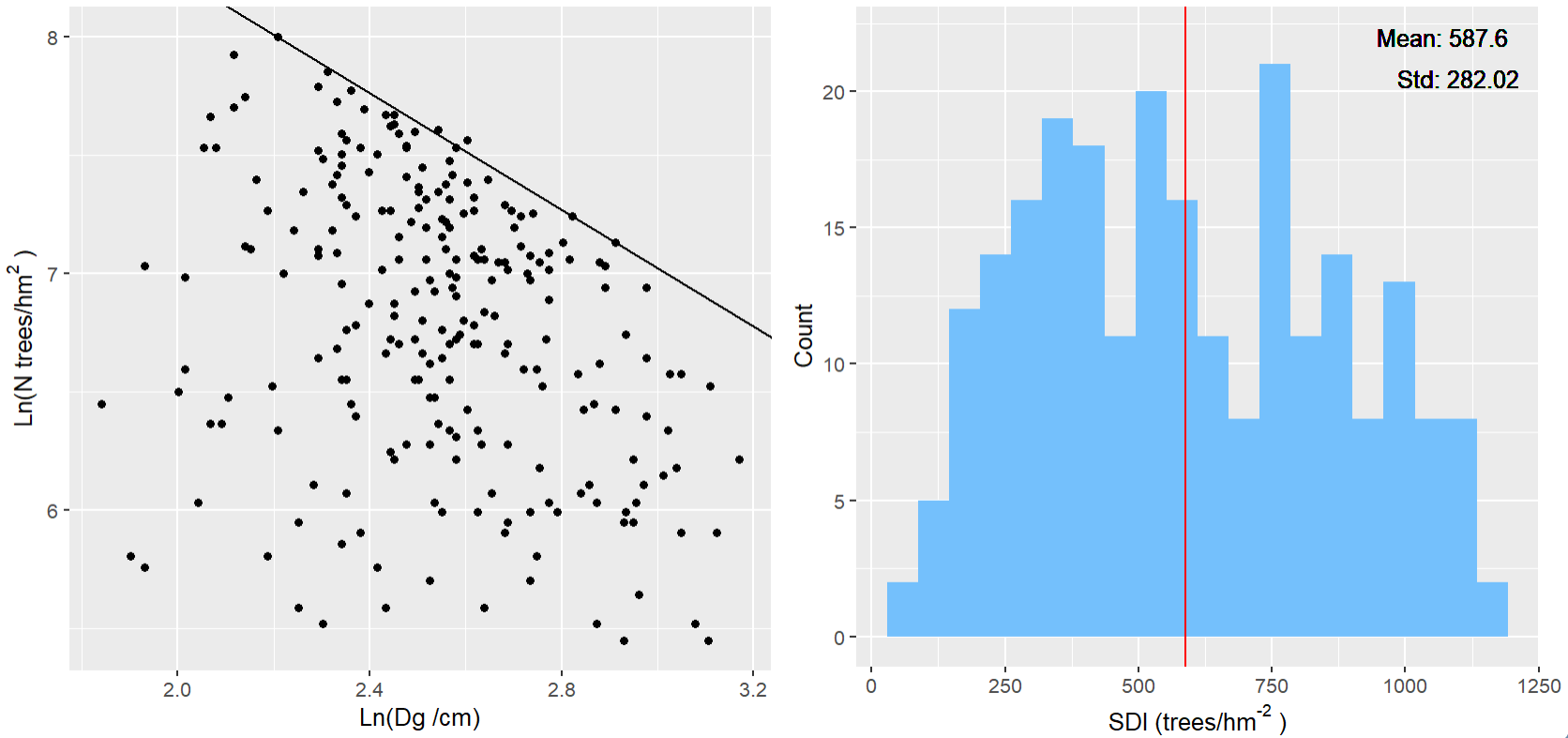


**Fig. S3** The fitted maximum size-density line (black; left) and the corresponding frequency distribution of stand density index (SDI; right) for natural *Larix* *gmelinii* forests, where N is the number of trees per hectare and Dg is the mean diameter at breast height; and the red line is the mean value of SDI.
